# Medicinal brandy^☆^

**DOI:** 10.1016/j.resuscitation.2011.03.005

**Published:** 2011-07

**Authors:** Henry Guly

**Affiliations:** Emergency Department, Derriford Hospital Plymouth, Derriford Rd., Plymouth, Devon PL6 8DH, UK

**Keywords:** History of resuscitation, Brandy, Alcohol

## Abstract

This paper describes the use of brandy and other forms of alcohol in the latter part of the 19th and early 20th centuries. Its prime use was as a cardiac stimulant as it seemed to increase the cardiac output and blood pressure. However it was also recognised as a depressant and was used as a sedative. Reconciling these two actions caused difficulties. In addition it was used as a food for invalids.

## 1 Introduction

Resuscitation during the latter part of the 19th century and early 20th century might include the use of brandy or other forms of alcohol. The literature of the Heroic Age of Antarctic exploration (1897–1922) contains many references to “medicinal” (or similar descriptions of) brandy. For example Wilson said of Scott's first expedition; “No alcohol was taken on sledge journeys, except for a small can of brandy for emergencies”^1^ and Ekelöf, doctor to the Swedish Antarctic Expedition (1901–1904), describing the use of spirits, said: “For the second year there were only very few bottles left, which were reserved for festive occasions or for medical use”.^2^ Spirits other than brandy could be used: on the Belgian expedition, Amundsen “gave Tollefsen a glass of cherry liqueur on the doctor's advice” for “exhaustion, mixed with madness”^3^ but other spirits were thought less useful. Thus Bernacchi on the Southern Cross expedition (1898–1900), in complaining that the expedition leader had consumed all the brandy, wrote in his diary: “Unless the doctor has a bottle or so, we have not a drop of brandy at Cape Adair for medicinal purposes. On this occasion we were obliged to use whiskey. It is really scandalous.”^4^ Brandy was in the British Pharmacopoeia (“Spiritus Vini Gallici”) and the belief in its superiority was not confined to lay people: the Lancet said that “…brandy is so universally regarded as superior to all other spirits from a medicinal point of view…”^5^ and “some hold that the stimulating and restorative effect is referable chiefly to the alcohol, but there can be no doubt that these effects are enhanced or diminished to a greater or less proportion of other bodies chiefly of the ether type”.^6^

When there were problems with the supply of brandy following infection of the vines with phylloxera, in the late 19th century, and other spirits were passed off as brandy, the Lancet set up a commission to examine the subject and concluded that “some control over the sale of substitutes for brandy should be established”^5^ and a pharmaceutical company provided brandy in ampoules.^7^ Old brandy was, generally, considered better than recently distilled spirit.^8^ Although the British Pharmacopoeia said that whisky (Spiritus frumenti) was often preferred to brandy because it is more readily obtained unadulterated,^9^ the Lancet had earlier warned that “A spirit derived exclusively from grain … will be less ethereal, if ethereal at all, than grape-derived spirit and *a priori* a less powerful restorative”. However the Lancet clearly accepted that whisky was a useful drug as varieties of whisky were reviewed in its pages.^10^

**Figs. 1 and 2:**
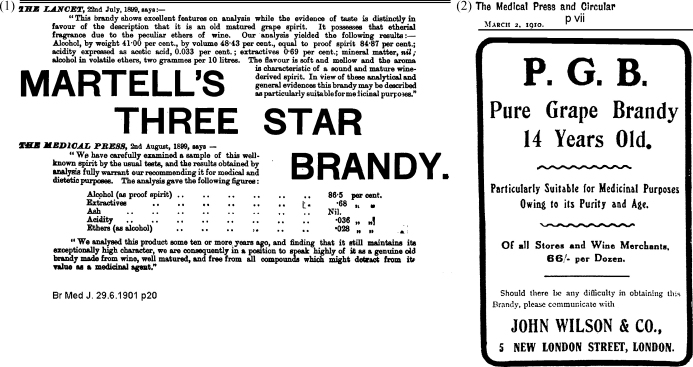
Advertisements for brandy from medical journals.

Brandy and whisky were advertised in medical and nursing journals (Figs. 1 and 2) and the same journals discussed their merits. Thus the British Medical Journal felt that Victorian Vineyards’ “brandy is a pure product and well worthy of the attention of the profession”^11^ and that “no other kind of brandy corresponds better to medical necessities than pure grape brandy. The “pure grape brandy” which Messrs. Canton and Co. … have sent to us is correctly so described…”.^12^ During the prohibition era when alcoholic drinks were banned in the USA from 1919, physicians lobbied strongly for the right to prescribe alcohol and a US survey in 1921 showed that 51% of physicians advocated prescribing whiskey and 26% believed that beer was “a necessary therapeutic agent.^13^ At St. Bartholomew's hospital feeding cups were kept on the anaesthetic trolleys for the administration of brandy in an anaesthetic emergency and these were still there as late as 1963^14^ though brandy had not been administered in living memory.^15^

The aim of this paper is to briefly describe the medical use of brandy and other forms of alcohol in the late 19th and early 20th centuries. I do not describe the social use (and misuse) of alcohol.

## 2 Use of alcohol

Alcohol has been both consumed as a beverage and used in medicine since time immemorial but by the end of the 19th century medicine had developed a scientific basis^16^ and although alcohol was not being used nearly as much as in earlier times, it still had an important role. There was a wide spectrum of views on its use in medicine. At one extreme were those who felt that as alcohol was a stimulant, it should be beneficial in all disease states. Wilkes quotes a London doctor who “gave brandy to all his cases, for he found all the Bermondsey people weak and required it”.^17^ At the other extreme was the temperance movement. Alcohol abuse caused (as it still causes) major medical and social problems and the prescribing habits of doctors, such as the one quoted above, were thought to contribute to this. In 1871 a statement from the British Medical Temperance Society said: “As it is believed that the inconsiderate prescription of large quantities of alcoholic liquids by medical men for their patients has given rise, in many instances, to the formation of intemperate habits, the undersigned, while unable to abandon the use of alcohol in the treatment of certain cases of disease, are yet of opinion that no medical practitioner should prescribe it without a sense of grave responsibility. They believe that alcohol, in whatever form, should be prescribed with as much care as any powerful drug, and that the directions for its use should be so framed as not to be interpreted as a sanction for excess, or necessary for the continuance of its use when the occasion is past”.^18^

The London Temperance Hospital (founded in 1873) discouraged the use of alcohol but did not forbid it. However they questioned its value. Ridge noted that “It is very easy to say that alcohol is very useful in the treatment of disease, and sometimes essential to save life, but how do they know it? How can they possibly tell unless they give nature an opportunity of showing what she can do in the absence of alcohol?... The fact that improvement often sets in after the use of alcohol is no proof whatever, for I have repeatedly seen sudden turns for the better in all kinds of cases treated without it”.^19^

## 3 Use as a stimulant

A problem for doctors was reconciling that brandy (the most commonly used form of alcohol) seemed to have both stimulant and sedative effects. However it is clear that the emergency use of brandy was as a stimulant: the British Pharmacopoeia of 1907 discusses alcohol rather than brandy specifically, and states “As a circulatory stimulant the value of alcohol is undoubted; it increases the output of blood from the heart, and slightly raises blood pressure... Its action may be due either to a direct stimulant effect on cardiac muscle, or to the fact that it affords a readily assimilable source of energy”.^20^ It had long been observed that if a patient fainted, a dose of brandy would aid their revival but this effect was too rapid to be due to a pharmacological effect and was clearly a reflex caused by stimulation of the nerves in the mouth.^17^

Dixon, in animal and human experiments, investigated the effect of alcohol on the heart rhythm, the cardiac output, the vessels and the blood pressure. Dilute alcohol had inconsistent effects on the heart rate but “when concentrated alcohol, 20 to 50 per cent, is taken by the mouth the pulse during the space of a minute or two is always accelerated; this is not an action peculiar to alcohol and it can be observed after the administration of any irritant such as mustard and water, or even after water alone if it is sufficiently hot.”^21^ However, “alcohol as a 50 per cent. solution was a more efficient “stimulant” than the other substances (mustard, essential oil, hot water) which were tried … it quickened the heart considerably. If the alcohol was retained in the mouth a few seconds only and was then spat out, the effect quickly passed off, but if it was taken into the stomach the acceleration was prolonged for half an hour or longer”. He demonstrated that this was not a pharmacological effect because “When the same amount of alcohol is given well diluted these effects are not seen.”^21^ When the heart was perfused with alcohol in low concentrations (0.1–0.2%), the cardiac output increased but at higher concentrations, it decreased. His findings on the effect on the blood pressure are at odds from those described in the pharmacopoeia “The cardiac output is increased and the sum-total effect on the vessels is rather towards dilatation than constriction, so that the blood-pressure may either rise or remain constant according to which of these two factors holds the balance”.^21^

While its value in neurogenic fainting was not doubted, opinions varied about its value in fainting as a result of haemorrhage as alcohol was said to inhibit clotting.^17^ Nevertheless it was used in severe haemorrhage, particularly obstetric haemorrhage^22,23^ but also in other forms of haemorrhage.^24–26^

Brandy was also given by injection,^27^ rectally^26,28^ and even intravenously. In the (successful) resuscitation of one patient with a ruptured ectopic pregnancy, “Eight and a half pints of hot saline solution with an ounce of brandy were injected”.^29^

For lesser conditions, tonics were much used as stimulants and alcohol was the basis of many of these,^30^ the alcohol concentration of which was often greater than that of wine and approached that of sherry or port.^31^

Alcohol was also used as a stimulant in hypothermia as the peripheral vasodilatation and feeling of warmth that it provoked was thought to be helpful, despite the fact that it was known that the vasodilation caused heat loss and worsened the hypothermia.^32,33^ Even now, when this complication is much better known, walkers and hunters take a hip flask of spirits to warm them on winter days.

Pharmacologically, alcohol is a depressant. The British Pharmacopoeia described it as a cardiac stimulant as described above but also says that the “benefits which are obtained from its use in various conditions known as nervous shock – conditions in which the brain may be already over-excited – are due to its depressant action and not, as has often been said, to a stimulant effect.”^20^ In 1920 the chairman of a symposium hoped that the meeting would “clear up the discrepancy, between this lack of any sound experimental evidence, that alcohol is a stimulant and its still widely prevalent use as a stimulant of depressed or failing respiration and circulation”. He concluded that “One must allow something … for the greater hold of tradition on clinical practice than on experimental science; but I am not satisfied to regard this as the whole explanation of the anomaly. I think a good deal of weight may be allowed to the consideration that the experimental results have been obtained on normal subjects, while clinical experience is dealing with functions depressed, not merely by actual weakness, but by inhibitions due to reflex action or to influences from the higher centres.” As an example: “If and when alcohol assists recovery from syncope due to fright or pain, I imagine that we have a condition in which the action of the heart and the vasomotor tone are subjected to severe central inhibition, and that alcohol helps to weaken and remove this inhibition”.^34^

## 4 Use in fevers

Alcohol was much used in pyrexial illnesses, especially pneumonia and typhoid^17^ and there were several reasons for its use. Firstly it lowered the temperature by its vasodilatory effect. Its depressant effects were also valuable; “in respiratory embarrassment, especially in the rapid, shallow, inefficient breathing of broncho-pneumonia, alcohol quiets the respiration, and so makes it more efficient,”^8^ thereby improving oxygenation. It also reduced delirium. Alcohol could supply up to 40% of a patient's required calories. It is easily absorbed and so was recommended for use in those with difficulty in absorbing food or those with anorexia and vomiting. “In acute fever … alcohol is an admirable food, because it requires no digestion and is easily absorbed”.^8^

## 5 Use as food

The British Pharmacopoeia said that “The most important action of alcohol is on metabolism; in ordinary doses it is almost completely oxidized, and spares the oxidation of fat … it surpasses starch and sugar in alimentary value, since weight for weight, it contains more energy.”^20^ When used as a food, there was a limit to rate at which alcohol could be metabolized so small quantities often were better (this also reduced the risk of intoxication). It does not ferment in the gut and so was useful in severe flatulence.^35^ In convalescence, apart from its calorific value, “certain patients are made more comfortable and contented, worry less and take their food with keener enjoyment, if they are given alcohol” or, rather, “alcohol in a form in which the patient enjoys it”. This is obviously not an effect of the alcohol itself as “it would hardly be maintained that the same quantity of the drug, administered in a distasteful mixture, would have the same effect.”^34^ This bedevilled the clinical research into the use of alcohol as “no person, whether actually ill or convalescent, is ever given pure alcohol and water” and some of the effects might be caused by “other bodies present in wines and spirits”^8^ Apart from its use as a drug, brandy (and other alcoholic drinks) were also included within the term “medical comforts”, a description of food and drink for the ill, injured and convalescing. Medical comforts also included beef extracts, soups, arrowroot and easily digested foods. However the borders between its use as a medicine, as a medical comfort and as a social lubricant blurred at times.

Before insulin, alcohol was also of use as a source of calories in diabetes, as a diet restricted enough to prevent glycosuria, might not provide enough calories to sustain life, and alcohol could be of great value.^36^

## 6 Use as a sedative

Not only could alcohol be used as a stimulant, it was also used as a sedative: “the chief therapeutical effect of alcohol in a beneficial sense is that it is a pleasant depressant, peculiarly efficacious in inhibiting peripheral impulses, such as pain here, and discomfort there, that it diminishes those trivial worries which bother the sick. In larger doses it has the advantage of inducing sleep.”^8^ Specifically it was useful in insomnia, especially in old people, and in cases of delirium and restlessness in acute illness^33^ and even in children: “Alcohol is, I suppose, the most valuable sedative and hypnotic drug we possess for infants and young children”.^37^

## 7 Other uses

Alcohol was also used by inhalation in anaesthesia to prevent the cardiac complications of chloroform^38^ and could also be used, pre-operatively for pre-medication. It was also used by inhalation for the treatment of heart failure^38^ and might be used to disguise the taste of castor oil and, perhaps, other unpleasant-tasting medications.^39^

Alcohol also stimulated the flow of gastric juices and its vasodilator effects were of benefit in angina.^35^

## 8 Decline

The use of alcohol gradually declined as a result of better understanding of pathology and the pharmacology of alcohol and better alternative treatments, though doubtlessly, some doctors took longer to give up prescribing it than did others. In haemorrhagic shock, the better understanding of shock that occurred during the First World War and the wider application of intravenous fluids led to brandy being abandoned though it was still mentioned (if not recommended) in relation to obstetric haemorrhage into the 1930s.^40,41^ In pneumonia, its use seems to have gradually faded as can be illustrated by quoting from articles in the medical press. In 1933 it was recommended as a sedative and as a food and was particularly useful to stem delirium tremens in alcoholics with pneumonia but not recommended as a routine.^42^ In 1936 it was felt useful for those who were “most urgently ill”.^43^ Another author, the same year, said that although alcohol had been the most commonly used drug in pneumonia, opposition was growing as it could worsen circulatory failure.^44^ By 1941 sulphonamides were available and in 1945 an author merely said that he had observed no harm from alcohol^45^ while by 1949, it was recommended that alcohol had no place in pneumonia other than in the avoidance of delirium tremens.^46^

## Conflict of interest statement

There are no conflicts of interest.

## References

[1] Wilson E.A. (1905). The medical aspect of the Discovery's voyage to the Antarctic. BMJ.

[2] Ekelöf E. (1904). Medical aspects of the Swedish Antarctic Expedition October 1901–January 1904. J Hyg.

[3] Decleir H. (1999). Roald Amundsen's Belgica diaries.

[4] Crawford J. (1998). That first Antarctic winter.

[5] Anon. (1902). Special analytical commission on brandy. Lancet.

[6] Anon. (1899). What is genuine brandy?. Lancet.

[7] Hallows S. (1905). Brandy sterules. Lancet.

[8] White W.H. (1920). Discussion on the value of alcohol as a therapeutic agent. Proc R Soc Med.

[9] British Pharmaceutical Codex Pub Pharmaceutical Society, London; 1907. p. 1076.

[10] Anon. (1900). Analytical records from the Lancet laboratory: highland malt whisky. Lancet.

[11] Anon. (1892). Reports and analyses and descriptions of new inventions in medicine, surgery, dietetics, and the allied sciences Victorian Vineyards brandy. Br Med J.

[12] Anon. (1894). Reports and analyses and descriptions of new inventions in medicine, surgery, dietetics, and the allied sciences: pure grape brandy. Br Med J.

[13] Appel J.M. (2008). “Physicians are not bootleggers”: the short, peculiar life of the medicinal alcohol movement. Bull Hist Med.

[14] Ballantine R.I.W. (1963). An old Barts custom. Br Med J.

[15] Hewer C.L. (1963). An old Barts custom. Br Med J.

[16] Bynum W.F. (1994). Science and the practice of medicine in the nineteenth century.

[17] Wilks S. (1891). Introduction to a discussion on the effects of alcohol. Br Med J.

[18] Ridge J.J. (1888). Medical declarations re alcohol. Br Med J.

[19] Ridge J.J. (1883). The treatment of disease without alcohol. Br Med J.

[20] British Pharmaceutical Codex; 1907, op cit p. 69.

[21] Dixon W.E. (1907). The action of alcohol on the circulation. J Physiol.

[22] Lawrence H.C. (1870). Remarks on post partum haemorrhage. Br Med J.

[23] Le Page J.F. (1883). On transfusion. Br Med J.

[24] Rattray A.M.T. (1892). Haemorrhage following tonsillotomy. Br Med J.

[25] Jessop E. (1893). Secondary haemorrhage after removal of the tonsils. Br Med J.

[26] Blenkinsop A.P. (1900). Report on three cases of gunshot wound. Br Med J.

[27] Turner H.M. (1905). The feeding of delirious patients. Br J Nurs.

[28] Cameron A.M. (1912). What care must be taken when removing the clothes of a patient badly burnt?. Br J Nurs.

[29] Whiteford C.H. (1899). A case of ruptured tubal pregnancy. Lancet.

[30] Anon. (1909). The composition of some proprietary food preparations. II. Tonic wines. Br Med J.

[31] Anon. (1909). The composition of some proprietary dietetic preparations. Br Med J.

[32] Anon. (1874). The action of alcohol. Br Med J.

[33] Kinsey R.H. (1883). An address on alcohol and on drainage. Br Med J.

[34] Dale H.H. (1920). Discussion on the value of alcohol as a therapeutic agent. Proc R Soc Med.

[35] Hutchison R. (1920). Discussion on the value of alcohol as a therapeutic agent. Proc R Soc Med.

[36] Leyton O. (1920). Discussion on the value of alcohol as a therapeutic agent. Proc R Soc Med.

[37] Harding M.E. (1920). Discussion on the value of alcohol as a therapeutic agent. Proc R Soc Med.

[38] Willcox W.H. (1920). Discussion on the value of alcohol as a therapeutic agent. Proc R Soc Med.

[39] Stewart A.V. (1897). Describe the most palatable method of administering a dose of castor oil. Nurs Rec Hosp World.

[40] Brown O’D. (1931). Shock following blood extravasation. Br Med J.

[41] Murray E.F. (1938). The obstetrical “flying squad”. Br Med J.

[42] Johnson R.S. (1933). The treatment of pneumonia. Postgrad Med J.

[43] Langley G.J. (1936). The treatment of lobar pneumonia. Postgrad Med J.

[44] Wynn W.H. (1936). The treatment of pneumonia. Br Med J.

[45] Howell T.H. (1945). Pneumonia in old age. Postgrad Med J.

[46] Scadding J.G. (1949). Management of acute pneumonia in adults. Br Med J.

