# Supplementary material for: Medicinal brandy
Source: Resuscitation. 2011 Jul;82(7-2):951–4. doi: 10.1016/j.resuscitation.2011.03.005 (PMC3117141; doi:10.1016/j.resuscitation.2011.03.005)
Supplement: Supplementary file 1 [file mmc1.doc]

Resumen

Este artículo describe el uso de Brandy y otras formas de alcohol en la última parte del siglo 19 y primera parte del siglo 20. Su primer uso fue como estimulante cardiaco ya que parecía estimular el gasto cardiaco y presión de sangre. Sin embargo también se reconoció como depresor y fue usado como sedante. El reconciliar estas 2 acciones causó dificultades. Además se usó como alimento para inválidos.

© 2011 Publicado por Elsevier Ireland Ltd.

*Palabras clave*: Historia de la resucitación; Brandy; Alcohol
